# Characterization of Volatile Components in *Makgeolli*, a Traditional Korean Rice Wine, with or without Pasteurization, During Storage

**DOI:** 10.3390/molecules18055317

**Published:** 2013-05-08

**Authors:** Hye-Jung Park, Sang Mi Lee, Sang Hoon Song, Young-Suk Kim

**Affiliations:** 1 Department of Food Science and Engineering, Ewha Womans University, Seoul 120-750, Korea; 2 CJ Foods R&D, CJ Cheiljedang, Seoul 152-051, Korea

**Keywords:** *makgeolli*, volatile components, odor-active compounds, unpasteurized, pasteurized, storage period

## Abstract

Changes in the volatile components of unpasteurized and pasteurized *makgeolli* during 30 days of storage were investigated by gas chromatography-mass spectrometry (GC-MS) and GC-olfactometry (GC-O). A total of 11 odor-active compounds such as 3-methyl-1-butanol (isoamyl alcohol), 2-methyl-1-butanol, 2,3-butanediol, butanoic acid, 3-methylbutanoic acid (isovaleric acid), 2-methylbutanoic acid, 3-(methylthio)-1-propanol (methionol), 2-phenylethanol, ethyl decanoate, ethyl dodecanoate, and ethyl tetradecanoate were determined in both the pasteurized and unpasteurized *makgeolli* during 30 days of storage. Although there were no significant differences in the concentrations of odor-active compounds at the initial storage time, most of odor-active compounds were more significantly increased in unpasteurized *makgeolli* compared to the pasteurized one during the storage period.

## 1. Introduction

The consumption of alcoholic beverages made from rice has been increasing in Asian countries such as Korea, Japan, and China. Among these beverages, there has been particular interest in *makgeolli*, a traditional Korean rice wine. *Makgeolli* has distinctive properties, mainly due to the rice fermentation process, such as a white or beige color, creamy texture, sweet and slightly carbonated flavor, and, in particular, its own characteristic odor note [1,2].

The fermentation of *makgeolli* usually uses starters such as molds (*Aspergillus*, *Rhizopus*, and *Mucor* spp.) and yeasts (*Saccharomyces*, *Pichia*, *Candida*, *Torulopsis*, and *Hansenula* spp.) [1,3]. Lactic acid bacteria (*Leuconostoc* and *Lactobacillus* spp.) can be also added to supplement the fermentation starter with the molds and yeasts to prevent contamination by other bacteria [1]. There are some naturally growing bacteria, such as *Micrococcus*, *Bacillus*, *Aerobacter*, and *Pseudomonas* spp., that can lead to spoilage of *makgeolli* [3]. All of these microorganisms influence the quality of the product—and, in particular, its volatile components—via several metabolic pathways including alcoholic and lactic acid fermentation [1,3,4]. Molds contain diverse enzymes such as amylase, glucoamylase, glucose oxidase, lipase, and protease [1,4], which can convert macromolecules including starch, proteins, and lipids into small molecules such as sugars, amino acids, and fatty acids that yeasts can use [1,4]. Alcoholic fermentation by yeasts can lead to the formation of diverse components including alcohols, esters, organic acids, fatty acids, and amino acids, which can themselves affect the quality of *makgeolli* [1,2,4].

Commercial *makgeolli* is distributed in two forms: heat pasteurized or unpasteurized. Pasteurization is a process that is used to treat many food products to extend their shelf-life and improve their microbial safety [3]. The shelf-life of *makgeolli*, which is a primary concern in the food industry, is affected mainly by the growth of molds, yeasts, and other microorganisms. Pasteurized *makgeolli* has a longer shelf-life because the pasteurization process eliminates yeasts, molds, and other microorganisms that produce gas or other by-products during storage and distribution [3]. Although *makgeolli* is generally treated using low-temperature, long-time (LTLT) pasteurization (rather than the high-temperature, short-time method), some changes in the physical, chemical, and sensory properties, including aroma and taste cannot be avoided during any heat treatment [3]. In particular, LTLT pasteurization was found to cause several problems, such as a strong off-flavor (burnt odor), discoloration, and layer separation [3].

Instrumental analyses have been applied in many researches for qualitative and quantitative determination of volatile components in diverse foods [5,6,7,8]. In particular, the analysis using gas chromatography (GC)-mass spectrometry (MS) for the identification and quantification of volatile or nonvolatile components in alcoholic beverages has been conducted extensively [9,10,11,12]. However, no previous study has investigated the volatile components and odor active compounds in *makgeolli*, including those responsible for the changes of flavor during storage.

The objective of the present study was to determine and characterize the volatile components of both pasteurized and unpasteurized *makgeolli*, including odor-active compounds, and the changes therein with storage time.

## 2. Results and Discussion

### 2.1. Odor-Active Compounds in Makgeolli

A total of 11 odor-active compounds were found in both the pasteurized and unpasteurized *makgeolli* during 30 days of storage: 3-methyl-1-butanol (isoamyl alcohol), 2-methyl-1-butanol, 2,3-butanediol, butanoic acid, 3-methylbutanoic acid (isovaleric acid), 2-methylbutanoic acid, 3-(methylthio)-1-propanol (methionol), 2-phenylethanol, ethyl decanoate, ethyl dodecanoate, and ethyl tetradecanoate. Table 1 lists their retention indices, odor descriptions, and aroma threshold values (ATVs).

Amyl alcohols (3-methyl-1-butanol and 2-methyl-1-butanol) have fermented and malt-like odor notes [13]. These are also detected in many other fermented alcoholic beverages such as wine, beer, and sake [6,13]. Although they can be related to positive aroma characteristics in wine at concentrations of less than 300 mg/L, they can mask the other aromas of wine at high concentrations [14].

Branched-chained organic acids (3-methylbutanoic acid and 2-methylbutanoic acid) have lower ATVs than their corresponding straight acid, butanoic acid, and are found in some fermented foods such as soybean paste [15,16]. In the present study, 3-methylbutanoic acid, 2-methylbutanoic acid, and butanoic acid were determined as odor-active compounds with unpleasant odor notes such as rancid and cheese-like. 3-(Methylthio)-1-propanol, which can be found in wine [14], was also detected in *makgeolli* as an odor-active compound with a vegetable-like odor note. Ethyl esters (ethyl decanoate, ethyl dodecanoate, and ethyl tetradecanoate) were detected as having floral and sweet odor notes.

**Table 1:** Odor-active compounds in *makgeolii*.

No.	RI ^a^	Components	Odor descriptions	ATV ^b^
1	737	3-methyl-1-butanol	fermented, malt, wine	300
2	739	2-methyl-1-butanol	fermented, malt, wine	300
3	786	2,3-butanediol	green, buttery	150,000
4	806	butanoic acid	cheese, unpleasant	173
5	844	3-methylbutanoic acid	fermented, cheese, rancid	33
6	852	2-methylbutanoic acid	fermented, cheese, rancid	100
7	978	3-(methylthio)-1-propanol	vagetable, onion	500
8	1115	2-phenethylethanol	floral, sweet, green	750–1,100
9	1394	ethyl decanoate	floral, honey	630
10	1590	ethyl dodecanoate	floral, honey	590
11	1788	ethyl tetradecanoate	floral, honey	400

^a^ RI; retention indices on DB-5MS column were determined using *n*-paraffins C_7_-C_22_ as external standards; ^b^ ATV; aroma threshold values of odor-active compounds in water (ppb) [16,17].

### 2.2. Comparison of Odor-Active Compounds in the Pasteurized and Unpasteurized Makgeolli during Storage

Table 2, Table 3 list the concentrations of odor-active compounds in the two forms (*i.e.*, pasteurized and unpasteurized) of *makgeolli* during the 30 days of storage, as evaluated using their relative peak areas on GC-MS chromatograms.

**Table 2:** The concentrations of odor-active compounds in unpasteurized *makgeolli* during storage.

No.	RI ^a^	Compounds	*m/z* ^b^	Unpasteurized *makgeolli* ^c^					ID ^d^
				0 day	5 days	10 days	20 days	30 days	
1	737	3-methyl-1-butanol	70	25.08 ± 0.91	52.40 ± 0.43	115.21 ± 57.94	89.42 ± 16.25	183.01 ± 9.95	A
2	739	2-methyl-1-butanol	70	0.39 ± 0.11	0.78 ± 0.03	2.65 ± 1.06	23.15 ± 37.59	269.70 ± 231.79	A
3	786	2,3-butanediol	57	38.18 ± 7.72	82.09 ± 6.30	221.18 ± 92.79	207.66 ± 2.46	359.27 ± 41.58	A
4	806	butanoic acid	60	0.01 ± 0.00	0.03 ± 0.00	0.16 ± 0.14	0.04 ± 0.00	0.09 ± 0.01	A
5	844	3-methylbutanoic acid	60	0.30 ± 0.05	0.80 ± 0.03	1.90 ± 0.97	1.61 ± 0.05	2.59 ± 0.05	A
6	852	2-methylbutanoic acid	74	0.38 ± 0.06	1.04 ± 0.04	2.17 ± 0.88	1.80 ± 0.17	3.09 ± 0.18	A
7	978	3-(methylthio)-1-propanol	106	3.26 ± 0.51	8.17 ± 0.50	17.78 ± 7.38	15.95 ± 0.46	21.49 ± 4.74	A
8	1115	2-phenethylethanol	91	32.99 ± 4.47	73.42 ± 4.20	168.53 ± 61.16	142.63 ± 4.66	280.28 ± 29.43	A
9	1394	ethyl decanoate	88	2.36 ± 0.56	5.57 ± 0.31	12.79 ± 4.27	10.51 ± 0.62	20.43 ± 0.82	A
10	1590	ethyl dodecanate	88	1.91 ± 0.70	4.21 ± 0.45	10.05 ± 5.99	9.84 ± 0.36	10.59 ± 0.76	A
11	1788	ethyl tetradecanoate	88	17.18 ± 8.62	27.43 ± 5.52	51.88 ± 35.77	77.91 ± 7.66	84.68 ± 6.09	A

^a^ Retention indices were determined using *n*-paraffins C_7_-C_22_ as external standards; ^b^ *m/z* for quantification of odor active compounds; ^c^ The value indicates the concentration of each component (n = 3) ± standard deviation using calibration curve and recovery tests; ^d ^Identification : A, mass spectrum and retention time were consistent with those of an authentic standard.

**Table 3:** The concentrations of odor-active compounds in pasteurized *makgeolli* during storage.

No.	RI ^a^	Compounds	*m/z*^ b^	Pasteurized *makgeolli* ^c^					ID^ d^
				0 day	5 days	10 days	20 days	30 days	
1	737	3-methyl-1-butanol	70	32.65 ± 2.17	51.24 ± 6.20	54.33 ± 1.92	55.78 ± 1.14	118.94 ± 7.22	A
2	739	2-methyl-1-butanol	70	0.78 ± 0.05	1.19 ± 0.24	1.58 ± 0.26	35.13 ± 59.53	2.13 ± 0.23	A
3	786	2,3-butanediol	57	46.04 ± 4.93	79.23 ± 4.99	97.87 ± 1.64	89.10 ± 12.31	131.83 14.11	A
4	806	butanoic acid	60	0.02 ± 0.00	0.03 ± 0.00	0.03 ± 0.00	0.03 ± 0.00	0.05 ± 0.00	A
5	844	3-methylbutanoic acid	60	0.43 ± 0.01	0.67 ± 0.04	0.64 ± 0.06	0.76 ± 0.06	1.41 ± 0.11	A
6	852	2-methylbutanoic acid	74	0.57 ± 0.03	0.90 ± 0.04	0.88 ± 0.03	0.99 ± 0.07	1.70 ± 0.04	A
7	978	3-(methylthio)-1-propanol	106	4.56 ± 0.10	7.05 ± 0.63	7.24 ± 0.35	8.03 ± 0.45	12.80 ± 0.97	A
8	1115	2-phenethylethanol	91	46.75 ± 2.39	71.94 ± 4.75	74.77 ± 2.94	82.82 ± 5.63	150.78 ±15.67	A
9	1394	ethyl decanoate	88	2.80 ± 0.07	4.49 ± 0.22	4.83 ± 0.21	5.04 ± 0.18	11.20 ± 1.03	A
10	1590	ethyl dodecanate	88	2.92 ± 0.04	4.07 ± 0.35	3.69 ± 0.22	4.00 ± 0.29	5.84 ± 1.27	A
11	1788	ethyl tetradecanoate	88	28.14 ± 2.14	40.38 ± 3.89	22.58 ± 5.39	27.85 ± 4.34	90.01 ± 24.55	A

^a^ Retention indices were determined using *n*-paraffins C_7_-C_22_ as external standards; ^b^ *m/z* for quantification of odor active compounds; ^c^ The value indicates the concentration of each component (n = 3) ± standard deviation using calibration curve and recovery tests; ^d ^Identification : A, mass spectrum and retention time were consistent with those of an authentic standard.

The concentrations of some odor-active compounds such as 3-methyl-1-butanol (isoamyl alcohol), 2-methyl-1-butanol, and 2-phenylethanol, which could be derived from the fermentation process, increased significantly in unpasteurized *makgeolli* during storage, are listed in Table 2. The amyl alcohols, 3-methyl-1-butanol and 2-methyl-1-butanol, can be formed by the fermentation process from isoleucine and leucine, respectively, through deamination and decarboxylation reactions during storage [6]. The increased concentrations of amyl alcohols could be related to the qualities of alcoholic beverages due to their characteristic fermented, malt-like, and alcoholic-like odor notes [6]. Higher alcohols, also known as fusel alcohols, which are formed by alcoholic fermentation, are important flavor components in alcoholic beverages. As mentioned above, higher alcohols can act as positive factors at concentrations lower than 300 mg/L, but as negative contributors at concentrations exceeding 400 mg/L [11]. Relatively high levels of these alcohols are considered as characteristic flavor components in some alcoholic beverages, such as whisky, traditional ales, and ciders, whereas in others, such as vodka and lagers, their presence is considered to be a defect [18].

Other alcohol components such as 2,3-butanediol and 3-(methylthio)-1-propanol were markedly increased in unpasteurized *makgeolli* during storage. 2,3-Butanediol, the presence of which is closely related to the quality of *makgeolli* due to its own characteristic odor note and slightly bitter taste, is formed by yeasts in the process of carbohydrate fermentation [19]. In the reductive decarboxylation step of citric acid fermentation, 2,3-butanediol can be formed via intermediate metabolites such as diacetyl and acetoin, which have buttery odor notes [19]. The present study could not detect the presence of diacetyl, which can be reduced to its corresponding alcohol (2,3-butanediol), probably because it was present at levels below the limits of detection. A previous study found that concentrations of diacetyl are usually lower than its ATV at the end of alcoholic fermentation due to reduction of diacetyl to acetoin and 2,3-butanediol, components that are less toxic to yeasts in wine [19]. 3-(Methylthio)-1-propanol is one of the volatile sulfur components, and has distinct odor notes such as cooked vegetable, boiled potato, and soup-like. It can be produced from methionine metabolism by yeasts through the fermentation process during storage. 3-(Methylthio)-1-propanol is considered to be one of the off-flavor components in wine and beer [20].

Butanoic acid, 3-methylbutanoic acid, and 2-methylbutanoic acid, which have unpleasant characteristic odor notes described as cheese-like, fermented, rancid, and acidic, can be important contributors to flavor changes in *makgeolli* during fermentation and storage. The concentration of butanoic acid increased until day 10 of storage in unpasteurized *makgeolli*, but significantly decreased thereafter, whereas it increased slowly throughout the storage period in pasteurized *makgeolli*. Butanoic acid can be converted from lipids by intracellular enzymes in lactic acid bacteria such as *Lactobacillus plantarum* [21]. According to Lee and Choi, the relative levels of butanoic acid decreased with increasing storage time during 16 days of fermentation because it reacted with ethanol and formed esters [2].

The relative concentrations of both 3-methylbutanoic acid and 2-methylbutanoic acid increased significantly in the unpasteurized *makgeolli*. Branched-chained amino acids such as valine, leucine, and isoleucine can be converted into branched-chained organic acids such as 3-methylbutanoic acid and 2-methylbutanoic acid by the fermentation process during storage [16]. 3-Methylbutanoic acid and 2-methylbutanoic acid can be formed from leucine and isoleucine catabolism processes, respectively, including oxidation and transamination [16]. The relative concentrations of 3-methylbutanoic acid and 2-methylbutanoic acid in unpasteurized *makgeolli* were greater than that of butanoic acid during storage, with the exception at day 10 (Table 2). However, throughout the storage period, the relative concentration of butanoic acid was higher than those of branched-chained butanoic acids in the pasteurized *makgeolli* (Table 3).

Levels of ethyl decanoate, ethyl dodecanoate, and ethyl tetradecanoate also increased considerably more in unpasteurized than in pasteurized *makgeolli* during storage. According to previous studies, yeasts can play a major role in the formation of diverse esters during fermentation process. Ethyl esters, which have pleasant odor notes such as floral, fruity, and perfume-like, have generally been reported to occur in other alcoholic beverages such as wine, beer, and sake [22]. During fermentation, the esterification of ethanol and organic acids occurs by yeasts, conferring fruity and floral odor notes upon alcoholic beverages [1,22]. It has been reported that the concentrations of ethyl esters depend on brewing parameters such as yeast strains, sugar content, fermentation temperature, and aeration [14]. Overall, it was possible to differentiate the *makgeolli* samples according to both pasteurization and storage time by assessing volatile components including diverse alcohols, acids, esters, and others.

## 3. Experimental

### 3.1. Samples

Seed culture was obtained by cultivating *Sacchromyces cerevisiae* (1 g) on koji (0.1 kg) with 0.13 L water at 24 ± 1 °C for 3 days. Then, koji (0.65 kg), *nuruk* (a traditional starter culture for brewing alcoholic beverage in Korea, 0.15 kg) [23], and water (4.4 L) were added to steamed rice (1.5 kg) before proceeding the first stage fermentation at 26 ± 1 °C for 2 days. The second stage fermentation was conducted at 26 ± 1 °C for 3 days after the addition of steamed rice (1.5 kg) with water (2 L). The final alcohol concentration of *makgeolli* was in the range of 15~17% after a sieving. For the pasteurized *makgeolli*, samples were heated at 65 °C for 20 min. All samples were sealed tightly in bottles and stored at cold room (4 °C) during storage. In each experiment, different bottles of *makgeolli* manufactured on the same day were used.

### 3.2. Chemicals

3-Methyl-1-butanol, 2-methyl-1-butanol, 2,3-butanediol, butanoic acid, 3-methylbutanoic acid, 2-methylbutanoic acid, 3-(methylthio)-1-propanol, ethyl decanoate, ethyl dodecanoate, ethyl tetradecanoate, sodium sulfate and n-alkane standards (C_7_-C_22_) were obtained from Sigma-Aldrich (St. Louis, MO, USA). 2-phenethylethanol and dichloromethane were purchased from Junsei Chemical Co., Ltd. (Nihonbashi-honcho, Tokyo, Japan) and J.T. Baker (Phillipsburg, NJ, USA), respectively.

### 3.3. Extraction of Volatile Components

Each 30 mL of unpasteurized or pasteurized *makgeolli* was mixed with re-distilled dichloromethane (60 mL) in a 200 mL bottle (PYREX, Thelenberg, Germany). Then, 2-ethyl-1-hexanol (100 µL, 1,000 μg/mL (w/v) in dichloromethane) was added as an internal standard, before mixed using a magnetic stirrer at 350 rpm and ambient temperature for one hour. After that, the mixture was centrifuged at 3,000 rpm, 4 °C for 10 minutes. The organic layer was filtered through anhydrous sodium sulfate (Na_2_SO_4_) on Advantec No. 2 filter paper (Advantec Toyo, Tokyo, Japan). Volatile components in organic extracts were isolated from non-volatile components using solvent-assisted flavor evaporation (SAFE) under high vacuum below 2 × 10^−5^ torr. The extracted volatile components were concentrated into a final volume of 0.3 mL (for GC-olfactometry) and 0.1 mL (for GC-MS), respectively, under a gentle stream of nitrogen gas.

### 3.4. Determination of Odor-Active Compounds by Gas Chromatography-Olfactometry

Gas chromatography-olfactometry (GC-O) was performed using an Agilent 7890A series gas chromatograph (Agilent, Palo Alto, CA, USA) equipped with flame ionization detector (FID), sniffing port ODP3 (Agilent, Palo Alto, CA, USA), and a DB-5MS capillary column (30 m length × 0.25 mm i.d. × 0.25 µm film thickness, J&W Scientific, Folsom, CA, USA). The GC temperatures were controlled as follows: 40 °C (5 min); 4 °C/min to 120 °C; 16 °C/min to 220 °C (10 min). One µL of the sample extract was injected in splitless mode and GC effluents were split to an FID and a sniffing port (1:1 ratio). The carrier gas was helium at a constant flow rate of 0.8 mL/min. The injector and detector temperatures were 230 °C and 250 °C, respectively.

### 3.5. Analysis by Gas Chromatography-Mass Spectrometry

One µL of SAFE extract was injected in splitless mode into DB-5MS capillary column (30 m length × 0.25 mm i.d. × 0.25 µm film thickness, J&W Scientific) installed in an Agilent 6890 N GC. The carrier gas was helium at a constant flow rate of 0.8 mL/min. The injector and detector transfer line temperatures were set at 230 °C and 250 °C, respectively. The oven temperature was programmed as follows: 40 °C (5min); 4 °C/min to 120 °C; 16 °C/min to 220 °C (10 min). The scan rate was programmed at 2.83 scans/sec and mass scan range was set at *m/z* = 35–550. An Agilent 5975 quadrupole mass spectrometer, connected to a GC, was used in electron ionization mode with ion source temperature set at 230 °C, analyzer temperature set at 180 °C and ionization energy set at 70 eV, respectively.

### 3.6. Identification and Quantification of Odor Active Compounds

The identification of volatile components including odor-active compounds were positively confirmed by comparing their retention times and mass spectral data with those of authentic standard compounds. The RI values of volatile components were calculated with n-paraffins from C_7_ to C_22_ as external standards.

For quantitative analysis, the relative concentrations of odor-active compounds in 2 types of *makgeolli* samples with and without a pasteurization were analyzed by GC-quadrupole MS after SAFE extraction. Each peak area was compared to that of the internal standard [100 μL of 2-ethyl-1-hexanol (w/v, 1,000 μg/mL)] in GC-quadrupole MS chromatograms using selective ion monitoring (SIM) mode. SIM was set to monitor *m/z* [70] for 3-methyl-1-butanol and 2-methyl-1-butanol, [57] for 2,3-butanediol, [60] for butanoic acid and 3-methylbutanoic acid, [74] for 2-methylbutanoic acid, [106] for 3-(methylthio)-1-propanol, [91] for 2-phenylethanol, [88] for ethyl decanoate, ethyl dodecanoate, and ethyl tetradecanoate. Calibration of each odor-active compounds was performed at six different concentrations of authentic standard compounds (w/v, 0.1–10,000 μg/mL). The recovery % for each odor active-compounds was obtained by addition of the mixed solution containing 11 standard compounds (w/v, 100 μg/mL). Concentrations of odor-active compounds were calculated on the base of calibration and recovery.

## 4. Conclusions

A total of 11 odor active compounds (3-methyl-1-butanol, 2-methyl-1-butanol, 2,3-butanediol, butanoic acid, 3-methylbutanoic acid, 2-methylbutanoic acid, 3-(methylthio)-1-propanol, 2-phenylethanol, ethyl decanoate, ethyl dodecanoate, and ethyl tetradecanoate) in *makgeolli* were analyzed quantitatively to determine the effects of pasteurization and storage time on them. In general, the relative concentrations of odor-active compounds showed a tendency to increase with increasing storage time in both pasteurized and unpasteurized *makgeolli*. However, the relative concentrations of these compounds (except for butanoic acid) increased more significantly in unpasteurized *makgeolli*. The results demonstrated that the quality of *makgeolli* could be significantly affected by pasteurization during the storage period.
